# Global dataset of species-specific inland recreational fisheries harvest for consumption

**DOI:** 10.1038/s41597-022-01604-y

**Published:** 2022-08-10

**Authors:** Holly S. Embke, Elizabeth A. Nyboer, Ashley M. Robertson, Robert Arlinghaus, Shehu L. Akintola, Tuncay Atessahin, Laamiri Mohamed Badr, Claudio Baigun, Zeenatul Basher, T. Douglas Beard, Gergely Boros, Shannon D. Bower, Steven J. Cooke, Ian G. Cowx, Adolfo Franco, Ma. Teresa Gaspar-Dillanes, Vladimir Puentes Granada, Robert John Hart, Carlos R. Heinsohn, Vincent Jalabert, Andrzej Kapusta, Tibor Krajč, John D. Koehn, Gonçalo Lopes, Roman Lyach, Terence Magqina, Marco Milardi, Juliet Nattabi, Hilda Nyaboke, Sui Phang, Warren M. Potts, Filipe Ribeiro, Norman Mercado-Silva, Naren Sreenivasan, Andy Thorpe, Tomislav Treer, Didzis Ustups, Olaf L. F. Weyl, Louisa E. Wood, Mustafa Zengin, Abigail J. Lynch

**Affiliations:** 1 U.S. Geological Survey, Midwest Climate Adaptation Science Center, 1954 Buford Ave, Saint Paul, MN 55108 USA; 2 Center for Limnology, University of Wisconsin – Madison, 680 Park St., Madison, WI 53706 USA; 3 Fish Ecology and Conservation Physiology Laboratory, Department of Biology and Institute of Environmental and Interdisciplinary Science, Carleton University, 1125 Colonel By Dr., Ottawa, ON K1S 5B6 Canada; 4 George Mason University, Department of Environmental Science and Policy, Department of Atmospheric, Oceanic, and Earth Sciences, 4400 University Dr., Fairfax, VA 24060 USA; 5 Leibniz Institute of Freshwater Ecology and Inland Fisheries, Department of Biology and Ecology of Fishes, Müggelseedamm 310, 12587 Berlin, Germany; 6 Humboldt-Universität zu Berlin, Faculty of Life Sciences, Division of Integrative Fisheries Management, Philippstrasse 13, Haus 7, 10115 Berlin, Germany; 7 Fisheries Department, Lagos State University, Lagos, Nigeria; 8 Fisheries Faculty, Fırat University, 23119 Elazıg, Turkey; 9 Water and Forest Department, Fisheries and Aquaculture Service, 3, Rue Harroun Errachid, Agdal, Rabat, Morocco; 10 Institute of Environmental Research and Engineering (UNSAM-CONICET, (1650) San Martin, Argentina; 11 Center for Systems Integration and Sustainability, Michigan State University, East Lansing, MI 48823 USA; 12 Gulf of Mexico Fishery Management Council, Tampa, FL 33607 USA; 13 U. S. Geological Survey, National Climate Adaptation Science Center, 12201 Sunrise Valley Drive MS 516, Reston, VA 20192 USA; 14 Centre for Ecological Research, Balaton Limnological Institute, H-8237 Tihany, Hungary; 15 Infinity, Social, and Ecological Solutions, 507 McLeod St., Ottawa, ON K1R 5P9 Canada; 16 University of Hull, International Fisheries Institute, Hull, HU6 7RX UK; 17 Instituto da Conservacão da Natureza e das Floresta, Avenida da República, 16, 1050-191 Lisboa, Portugal; 18 Dirección General de Investigación Pesquera en el Pacífico Norte, Instituto Nacional de Pesca, Pitágoras núm 1320, Col. Santa Cruz Atoyac, Deleg, Benito Juárez, DF 03310 México; 19 Autoridad Nacional de Acuicultura y Pesca de Colombia - AUNAP – Carrera 13 #40b -74, Bogotá, D.C. Colombia; 20 Kyung Hee University, College of Hotel & Tourism Management, 26 Kyunghee-daero, Dongdaemun-gu, Seoul, 130-701 Republic of Korea; 21 Global FlyFisher, Bogotá, D.C. Colombia; 22 Myanmar Fly Fishing Project, Yangon, Myanmar; 23 Stanisław Sakowicz Inland Fisheries Institute, Department of Ichthyology, Hydrobiology and Aquatic Ecology, Oczapowskiego 10, 10-719 Olsztyn, Poland; 24 Slovak Union of Anglers, Žilina, Slovakia; 25 Applied Aquatic Ecology, Arthur Rylah Institute for Environmental Research, Department of Environment, Land, Water and Planning, 123 Brown St., Heidelberg, Victoria 3084 Australia; 26 The Institute for Evaluations and Social Analyses (INESAN), Sokolovská 351/25, 186 00 Prague 8, Czech Republic; 27 Zimbabwe Parks and Wildlife Management Authority, P.O. Box CY140, Causeway, Harare, Zimbabwe; 28 Fisheries New Zealand - Tini a Tangaroa, Ministry for Primary Industries - Manatū Ahu Matua, 34-38 Bowen Street, 6011 Wellington, New Zealand; 29 Southern Indian Ocean Fisheries Agreement (SIOFA/APSOI), c/o DAAF, Bâtiment B, Parc de la Providence, 97489 Saint-Denis, France; 30 Makerere University, Department of Zoology, Entomology and Fisheries Sciences, P.O. Box 7062, Kampala, Uganda; 31 Kenya Marine and Fisheries Research Institute. Freshwaters Systems Directorate, P.O. Box 1881-40100 Nkuruma Rd, Kisumu, Kenya; 32 The Nature Conservancy, London, UK; 33 Department of Ichthyology and Fisheries Science, Rhodes University, Makhanda, South Africa; 34 MARE -Marine and Environmental Sciences Centre - Faculdade de Ciências, Universidade de Lisboa, 1749-016 Lisboa, Portugal; 35 Centro de Investigación en Biodiversidad y Conservación, Universidad Autónoma del Estado de Morelos. Av. Universidad 1001, Col. Chamilpa, Cuernavaca, CP- 62209 Morelos Mexico; 36 Wildlife Association of South India, 19 Victoria Road, Bengaluru, 560047 Karnataka India; 37 Centre for Blue Governance, University of Portsmouth, Portsmouth, PO1 2UP UK; 38 Department of Fisheries, Apiculture, Wildlife Management and Special Zoology, Faculty of Agriculture, University of Zagreb, Svetošimunska 25, 10000 Zagreb, Croatia; 39 Institute of Food Safety, Animal Health and Environment - “BIOR”, Lejupes 3, Riga, LV-1076 Latvia; 40 DSI/NRF Research Chair in Inland Fisheries and Freshwater Ecology, South African Institute for Aquatic Biodiversity, Makhanda, South Africa; 41 Central Fisheries Research Institute, Department of Fisheries Management, Trabzon, Turkey

**Keywords:** Freshwater ecology, Ecosystem services

## Abstract

Inland recreational fisheries, found in lakes, rivers, and other landlocked waters, are important to livelihoods, nutrition, leisure, and other societal ecosystem services worldwide. Although recreationally-caught fish are frequently harvested and consumed by fishers, their contribution to food and nutrition has not been adequately quantified due to lack of data, poor monitoring, and under-reporting, especially in developing countries. Beyond limited global harvest estimates, few have explored species-specific harvest patterns, although this variability has implications for fisheries management and food security. Given the continued growth of the recreational fishery sector, understanding inland recreational fish harvest and consumption rates represents a critical knowledge gap. Based on a comprehensive literature search and expert knowledge review, we quantified multiple aspects of global inland recreational fisheries for 81 countries spanning ~192 species. For each country, we assembled recreational fishing participation rate and estimated species-specific harvest and consumption rate. This dataset provides a foundation for future assessments, including understanding nutritional and economic contributions of inland recreational fisheries.

## Abstract (summary)

**Table:** 

Measurement(s)	inland recreational fisheries harvest
Technology Type(s)	Module 4: Literature References
Sample Characteristic - Organism	Osteichthyes
Sample Characteristic - Environment	inland waters
Sample Characteristic - Location	Global

## Background & Summary

Global analyses have estimated ~220–700 million people engage in recreational fishing globally^1^ and capture 40 billion fish per year^2^ in an industry valued at ~US$190 billion annually^3^. Inland recreational fisheries, defined as primarily leisure-driven fishing in lakes, rivers, and other landlocked waters, are critical to human livelihoods, health and wellbeing, and other ecosystem services worldwide. Recreational fishing in inland waters occurs throughout the world and constitutes the dominant use of inland fishes in many high-income regions^1,4^. Further, there is increasing interest in building the recreational fishing industry in developing regions for economic growth through ecotourism^5,6^.

Fish play a vital role in underpinning food security in many countries and communities as they provide a source of valuable nutrients important for healthy diets^7,8^. However, the contribution of recreationally harvested fish to the global food supply is largely unknown. Although catch-and-release is practiced in some recreational fisheries, in many fisheries the harvest and consumption of captured fish may contribute to food security, especially in lower-income regions or communities^9^, and increasingly in urban settings^10^. However, the contribution that inland recreational fisheries provide as a source of food remains unclear due to disparate and missing data^9,11,12^. Beyond global harvest estimates spanning large areas and select species (where available), few studies have explored comprehensive species-specific harvest patterns, although this variability has great implications for biodiversity management^13^ and nutritional security^8^. Accurately quantifying harvest and consumption at the species level is critical to understand how inland recreational fisheries contribute to the global food supply and how their role may be affected by global change.

The limited amount and availability of data related to inland recreational fisheries has hindered our ability to estimate its contribution to the food supply. One aspect of recreational fisheries that has been the subject of recent studies is participation rate, or how many people engage in regional fishing (e.g.^1,6,14^). These data provide a foundation for understanding broad-scale recreational fisheries patterns. Beyond participation rate, other key metrics, including harvest, species composition, and per capita consumption, are sparse, especially for the growing recreational fishing sector in the developing world, but when compiled may add insight into global patterns related to inland recreational fisheries^6,9^.

Here, we present a dataset that can help address a substantial knowledge gap in understanding the critical role that inland recreational fisheries serve in the global food supply. Based on a comprehensive literature search and expert knowledge review, we quantified multiple aspects of recreational fisheries for 81 countries, including ~192 species. For each country, we collated information on recreational fisher participation rate (%) and estimated species-specific inland recreational harvest (kg), species composition of harvest (%), and species-specific per capita consumption rate (kg per person). We targeted the search and review to harvest for consumption. We define harvest as retained catch excluding any released fishes and consumable biomass as the portion of harvest considered edible based on literature fillet yields. We acknowledge that not all harvested species may be consumed as regulations may require harvest for other purposes, for example in the case of non-native European carp in Australia, however for our purposes, we assume most harvested fishes are consumed.

This dataset provides a global perspective of inland recreational fisheries harvest and consumption as well as a foundation for a wide variety of future assessments, including understanding nutritional and economic benefits gained through inland recreational fisheries at different spatial scales. These data can also provide updated insights about inland recreational fisheries harvest and consumption while examining this fisheries sector as a coupled human-natural system, which is particularly important in the context of global change.

## Methods

### Country selection

To select countries for inclusion in our dataset, we consulted three recent studies that confirm national participation in inland recreational fisheries, globally (i.e., 1, 6, 14). Additionally, we consulted the Organization for Economic Cooperation and Development high income country lists to identify countries with relevant recreational fishing activities not included in the studies consulted. We excluded large ocean states due to their limited inland water surface area^15^. Finally, we called upon a curated panel of global recreational fisheries experts to review our final country list and confirm that we included all nations where inland recreational harvest could be estimated. Our final list included 81 countries from every inhabited continent encompassing high, middle, and low-income nations.

### Data collection

**Fig. 1:**
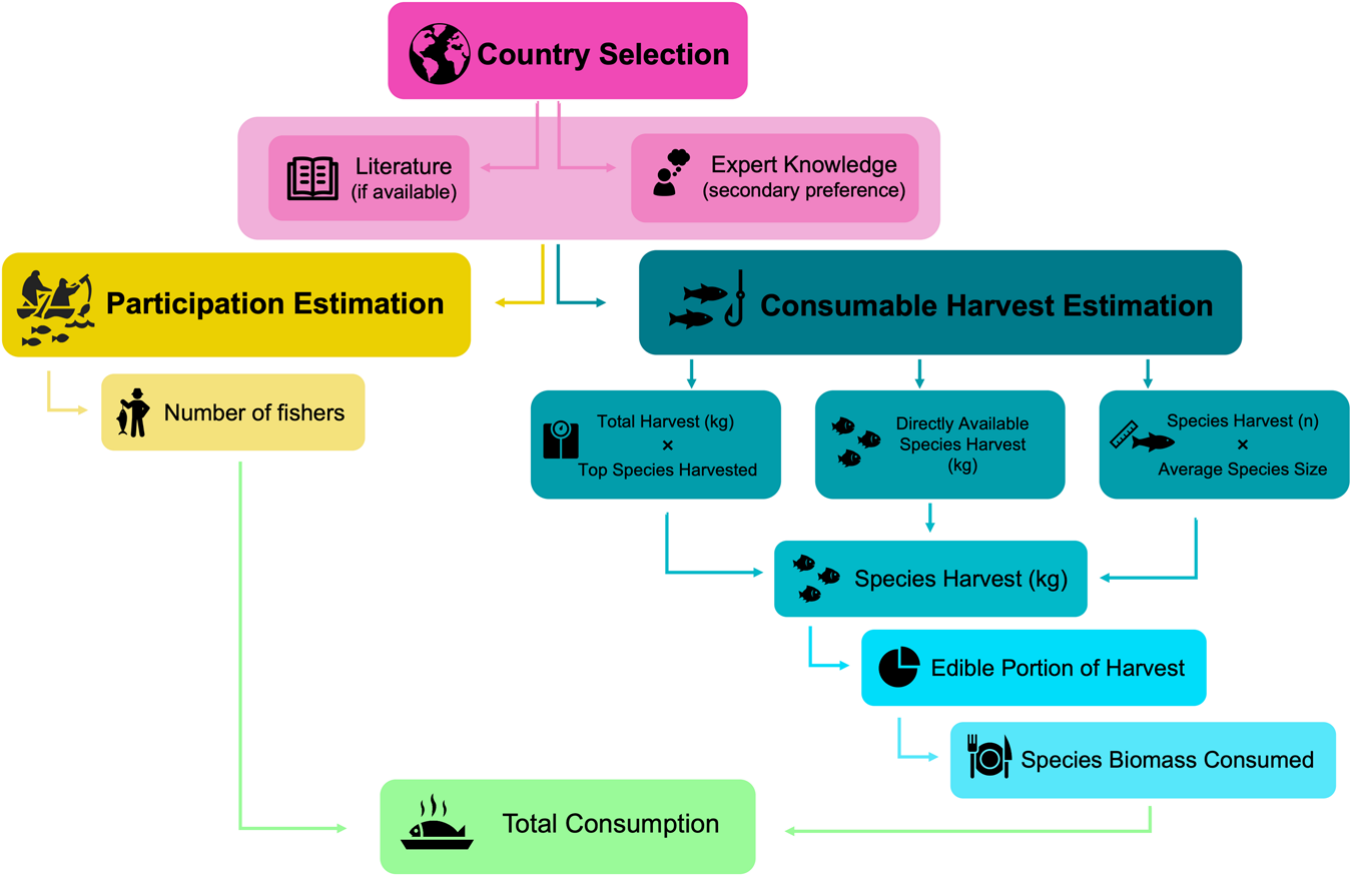
Conceptual diagram illustrating the methodology used to estimate global inland recreational fisheries participation rate, species-specific consumable harvest, and total fisher consumption for 81 countries.

Data on angler participation rate, species-specific harvest (kg or fish number), and species-specific consumption rate from inland recreational fisheries were targeted for each country in our dataset via literature searches (primary and grey literature), by accessing online governmental and Food and Agriculture Organization of the United Nations (FAO) databases, and by consulting individuals with expert knowledge in their respective country (Fig. 1). We used the Google translate tool to interpret documents written in languages when a native speaker was not available for consultation. For countries where data were not available, individuals with fisheries expertise in each country were identified via web searches and contacted via email with a detailed description of the required information. Follow up teleconferences or online meetings were conducted to clarify our request when needed. Experts generally responded to these requests in one of three ways: 1) by sharing data or reports that they had access to but were not previously available to the public (e.g., internal government reports - grey literature); 2) by indicating that no formal data or reports exist but providing expert-informed estimates based on experience, consultation with peers, and other informal knowledge sources; or 3) by indicating that no formal data or reports exist with no possibility of providing expert-informed estimates.

### Participation rate estimation

**Fig. 2:**
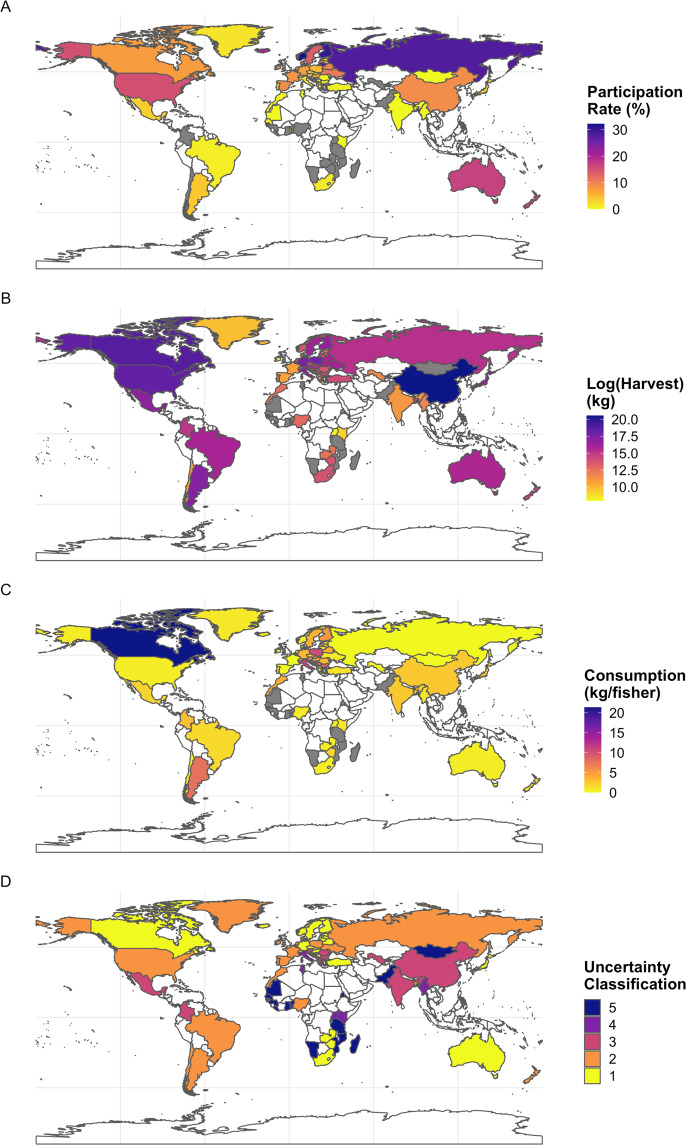
Global distribution of participation rate (%; panel A), log_e_(biomass harvest) (kg; panel B), per fisher consumption (kg/fisher; panel C), and uncertainty classification (panel D) for inland recreational fisheries. Darker colors indicate higher values for all metrics. Gray indicates no information was available for a given country for which we attempted to assess harvest, while white corresponds to countries we did not assess due to no relevant recreational fishing activities and data limitations.

Recreational fishing participation, defined as the portion of the population taking part in leisure-driven fishing activities, has previously been summarized for many of the countries of interest^1,3,14,16–18^. Therefore, the number of recreational fishers was preferentially estimated according to available literature sources (n = 50) and when not available (n = 13), expert knowledge provided participation rates (Figs. 1, 2A). In select instances (n = 18), no participation data were available. Comprehensive literature and expert knowledge references are provided in the raw version of the dataset. All country population sizes were provided by the CIA World Factbook 2020 (https://www.cia.gov/the-world-factbook/).

### Recreational harvest estimation

Hierarchical approaches were used to estimate total inland recreational harvest (kg) depending on information available for each country (Fig. 1). For most countries (n = 45), some form of inland recreational harvest information was available. If species-specific harvest estimates (kg) were available (n = 16), we summed these data to estimate total inland recreational harvest. The species represented included all those primarily targeted by recreational fishers for consumption. In some cases (n = 7), species-specific harvest (abundance) was known. We used corresponding literature-based mean total length (cm) and length-weight relationships to convert the number of fish harvested to biomass of fish harvested. The species-specific mean total length and length-weight relationship coefficients used are provided in the raw version of the dataset. In some cases (n = 22), angler harvest estimates were available for limited portions of a given country. For example, in Poland, Czarkowski *et al*.^19^ estimated angler species composition (%) and a mean annual catch of 46.1 kg per angler, 50% of which was harvested. Arlinghaus *et al*.^1^ provided an estimate of 1,996,800 Polish anglers, so harvest rates indicated in Czarkowski *et al*.^19^ were assumed to apply to the whole nation, thus total annual harvest was estimated at 46,026,240 kg in our dataset. When no recreational harvest information was available for a country but species harvest contributions were available (n = 15), we used a ‘nearest neighbor’ approach, wherein we applied the harvest rate (kg/angler or kg/ha) from the country geographically nearest to the country of interest. For instance, Colombian species harvest contributions were indicated by expert knowledge, but no harvest data were available. We therefore applied a nearest-neighbor harvest rate using Brazil’s estimates of ~0.14 kg of fish harvested per ha of freshwater surface area. Using the total freshwater surface area of Colombia (23,976,700 ha), we estimated 3,356,738 kg of fish harvested annually. We acknowledge this extrapolation approach does not account for variability in population density or cultural variability between countries. If no other information was available, we relied on expert knowledge to estimate an angler harvest rate (n = 6; Table 1). In countries such as Argentina, the number of fishers and estimated consumption were obtained using a regional perspective according to the type of fisheries and associated target species by water body characteristics (e.g., large temperate rivers, Patagonian lakes and rivers, shallow temperate lakes and temperate reservoirs). Demographic information from urban centers near these water bodies was used as a basis to estimate the percentage of recreational fishers, including an additional proportion of fishers coming from distant cities. The magnitude and spatial distribution of harvest are shown in Fig. 2B.

**Table 1:** Uncertainty classifications, number of countries, description of extrapolation approach, and an example of a country where that approach was used to estimate harvest and species contributions.

Uncertainty Category	Countries (N)	Extrapolation Approach	Example
1	23	Empirical harvest and species information available.	*Canada:* species-specific recreational harvest abundance estimates available. Species-specific length-weight relationships used to convert estimates to biomass harvested.
2	22	Limited information used to scale-up to the entire country.	*Poland:* Czarkowski *et al*.^19^ estimate a mean annual catch of 46.1 kg per angler for the country, 50% of which is harvested. There are 1,996,800 anglers, totalling 46,026,240 kg harvested.
3	15	Neighboring country information supplemented available information to make estimates.	*Colombia:* species information was indicated by personal communication and a report from Autoridad Nacional de Acuicultura y Pesca-AUNAP, but no empirical harvest data was available. Therefore, we assumed a nearest neighbor (Brazilian) harvest rate (~0.14 kg/ha). Based on the total freshwater surface area of Colombia (23,976,700 ha), we estimated 3,356,738 kg harvested annually.
4	6	No empirical harvest data available, but estimates made based on expert knowledge. Some species information available.	*Myanmar:* estimates very low harvest, but no data available (Vincent Jalabert, Myanmar Fly Fishing Project, oral communication, 2021), therefore assuming low harvest angler rate (~2 kg/angler), we estimated a total of 120,000 kg. harvested.
5	15	No data or information available - no estimates made.	*Benin:* no information available (as indicated by an expert).

Individual categories for each country are available in the raw dataset.

### Species estimation

**Fig. 3:**
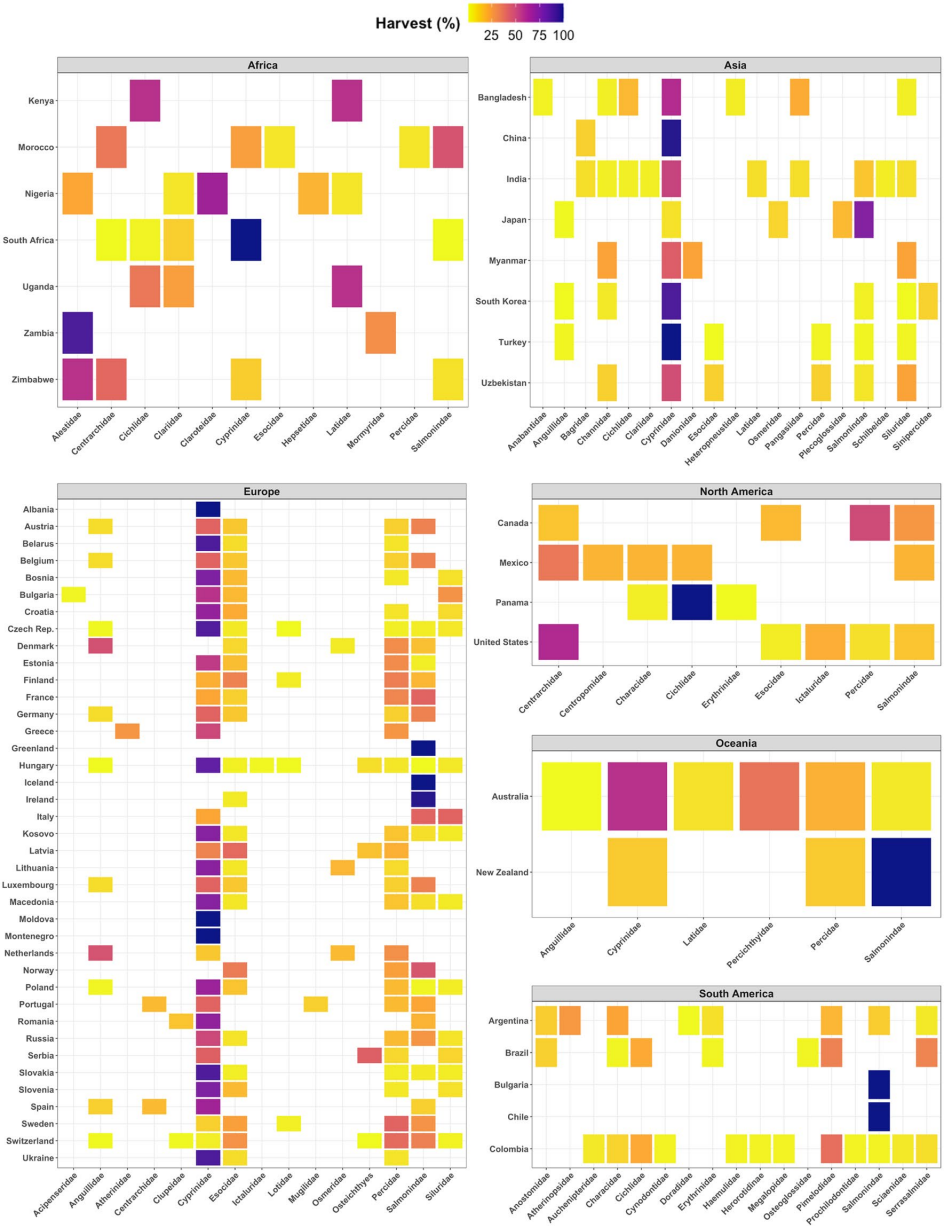
Scientific family composition of biomass harvest (%) for countries around the world. Darker colors indicate a higher percentage of total harvest for a given family and panels correspond to continents.

The predominant species harvested were identified from literature sources and/or expert knowledge. We quantified this information as the percent contribution of each species to the overall harvest estimate (Fig. 3). When species-specific contributions were unknown, we assumed an equal contribution of each species to overall harvest. We acknowledge that some species incorporated in the dataset may occur in both freshwater and marine environments depending on life history stage, however our aim was to estimate harvest of those individuals in freshwaters.

### Consumption estimation

Using total recreational harvest (kg) and species composition (%), we calculated species-specific harvested biomass for each country. All harvested fish were assumed to be consumed unless explicitly told otherwise; therefore, we recognize this assumption may introduce some additional uncertainty to our estimates. We used species-specific estimates of literature-based fillet yield (i.e., edible portion (%) of a given fish) to calculate the consumable portion of harvest (kg). We divided the consumable harvest (kg) by the number of fishers of the country to estimate per fisher consumption (Fig. 2C). We recognize that fisher consumption rate does not account for all who may be consuming the harvested fishes (e.g., family members of fishers), but feel this metric best represents the population consuming the fishes. In select cases when no participation information was available for a given country (n = 7), we used the participation rate from the nearest country geographically to estimate the number of fishers.

For each country, the data source for participation rate, harvest, species harvest contribution, and edible portion can be found in the raw version of the dataset. All harvest and species-specific estimates correspond to the most recent information available, with the majority (83%) of information from 2010–2021.

### Uncertainty classification

To account for variation in the quality of the data used to estimate harvest and species contributions, we developed an uncertainty classification which was applied to each country based on the degree of confidence we had in the data (Fig. 2D, Table 1). The five different classifications included: Category 1 - countries where species-specific harvest data (kg or n) were available (n = 23); Category 2 - countries where some information was available (e.g., estimates from part of a country) but some extrapolation approach was needed to ‘scale-up’ to the entire country-level (n = 22; see Poland example above in *Recreational harvest estimation* for more detail). Category 3 - countries where a nearest-neighbor approach was used to supplement available data for a given country (n = 15; see Colombia example above in *Recreational harvest estimation* for more detail). Category 4 - countries where no empirical harvest data were available, but estimates were made based on expert knowledge and some species information was available (n = 6). Category 5 - countries where no information was available (confirmed via literature sources or through expert knowledge; n = 15).

## Data Records

The inland recreational fisheries harvest and consumption dataset is provided as a CSV file in two forms: raw and formatted. The raw dataset contains all data, including species-specific length-weight coefficients and corresponding references (primary literature references provided in the refs. ^20–58^), and is intended as an original complete reference dataset. The formatted dataset is intended for re-use and contains a simplified version of the same data without references and length-weight coefficients. Each row in the datafile represents one species for a given country and each column represents an estimate or component. A full description of each column and its units or format is provided as a .xml metadata file, which is intended to extend the reuse potential of the dataset with full variable explanations. The raw and formatted datasets and accompanying metadata are freely available to the public supported by the U.S. Geological Survey (USGS) National Climate Adaptation Science Center^59^ (10.5066/P9904C3R).

## Technical Validation

The literature sources, online data archives, and expert information used to estimate country-level harvest and consumption rates in our dataset have been reviewed and validated in multiple ways. Each literature and online data source was reviewed at two levels: 1) an initial inspection that the required information (e.g., harvest data, species contribution) was available, and 2) a detailed review to extract information to make relevant estimates. When possible, estimates derived purely from expert knowledge were error checked by a second and sometimes third expert; however, low email response rates prevented this in 4 out of 6 instances. The data included in each entry (i.e., dataset row) was then reviewed by multiple authors of the dataset, including members of the curated panel of experts that were most familiar with the geographic region of a given country.

The USGS, an affiliation of multiple authors, requires internal review of science products, including manuscripts for publication and data incorporated into publications. The dataset was additionally reviewed and made publicly available through a Data Sharing Policy required of all USGS science products, and the manuscript was reviewed internally following Fundamental Science Practices. The USGS review process is intended to ensure and enhance the quality, accuracy, and availability of all science products released to the public and scientific community.

The sources included in the dataset were derived through a literature review that adopted rigorous and transparent methods as well as employed personal communication with experts, and information included in the dataset has been intensively reviewed. Although our literature and data search were as thorough as possible, it is still unlikely to be exhaustive or complete. First, despite our attempts to include non-English language documents, we are still likely to have missed available data due to language bias^60^. Second, we were unable to access data from some countries despite having knowledge that the required data likely existed due to lack of response from in-country experts. Third, it is likely that many participation and consumption values in our dataset represent underestimates given the ubiquitous under-reporting and the highly dispersed and informal nature of many inland recreational fisheries around the world^61^. We intend to continue updating the data as new information becomes available and request support from researchers and managers to fill gaps to improve accuracy.

## Usage Notes

The species-specific harvest and consumption dataset can be used to interrogate a wide array of scientific questions and provide valuable insights about the socio-ecological dynamics of inland recreational fisheries at a global scale. This dataset can also be combined with existing ecological, economic, and spatial datasets to enhance analyses. Questions of interest may include:

### Fishery benefits and value

1. What is the economic value of inland recreational fisheries consumption across varying geographies?
2. What are the social, economic, and environmental costs of replacing recreational fisheries with alternative food products?
3. What are the nutritional benefits to maintaining high biodiversity of recreationally-targeted inland fish?
4. How does inland recreational fisheries consumption relate to fish stocking practices across varying geographies?

### Fishery demographics

5. How does inland recreational fisheries consumption relate to fishers’ socio-economic status?
6. What is the relationship between inland recreational fish consumption and the poverty rate of a country or area?

### Data uncertainty

7. How does uncertainty in inland recreational fisheries harvest data influence global consumption estimates?
8. What are critical data gaps in the recreational fishery sector? (i.e., where could improved monitoring, reporting, and management better support sustainable recreational fisheries into the future?)

### Future impacts

9. What are potential effects of climate change on the status of stocks and related consumption and nutritional benefits of recreationally-targeted inland fishes?
10. What are the impacts of other major threats (e.g., habitat degradation) on recreationally-targeted inland fish consumption?

## Data Availability

No custom code was used to process or analyze the data.
